# Family support in the management of diabetes patients’ perspectives from Limpopo province in South Africa

**DOI:** 10.1186/s12889-022-14903-1

**Published:** 2022-12-23

**Authors:** M. H. Mphasha, T. M. Mothiba, L. Skaal

**Affiliations:** 1 Department of Human Nutrition and Dietetics, University of Limpopo, P/bag X1106, Sovenga, Polokwane, 0727 South Africa; 2 Faculty of Healthcare Sciences Executive Dean’s Office, University of Limpopo, Polokwane, South Africa; 3 Research office, Durban University of Technology, Durban, South Africa

**Keywords:** Patients living with diabetes, Family members, Support

## Abstract

**Background:**

Family support is linked with improved diabetes outcomes, whereas lack thereof is associated with complications. Living together with people in the same household doesn’t guarantee support in diabetes management. Hence it is critical to comprehend patient’s lived experiences regarding family support.

**Objectives:**

To explore family support in diabetes management.

**Methodology:**

Qualitative method and phenomenological exploratory descriptive design were used to collect data from 17 patients with 6 months or more living with diabetes and getting treatment in clinics of Senwabarwana, Limpopo province. Purposive sampling was used to select participants. One-on-one interviews were conducted using voice recorders and field notes for non-verbal cues were observed. Unstructured interview guide with principal question which enabled probing was used. Data were analysed using 8 Steps of Tesch’s inductive, descriptive, and open coding technique. Trustworthiness was ensured.

**Results:**

Participants get support from family members with regards to food, exercise, and collection of medication. Diabetic men with sexual dysfunction also reported getting similar support from their wives whereas, in contrary diabetic women do not get sufficient supports from their husbands. Loss of income among diabetic men has been reported as a source of martial conflicts and unsatisfactory support from wives.

**Conclusion:**

Family members of diabetic patients collect medication for patients, including cooking and serving meals to them. Wives of diabetic men outpace husbands of diabetic women in responsiveness to the health needs of their partners, respectively. Diabetic men with impaired sexual function get support from their wives, whilst female patients do not get similar support from their husbands. On the other hand, patients who lost income get inadequate spousal support, which compromises diabetes management and adherence to treatment. This study identifies gender disparities in spousal support as crucial issue requiring an urgent attention, hence the need for gender-biased family-centred diabetes care.

## Background

Larger part of diabetes care occurs within households, where patients reside, and this influences diabetes management behaviours [1]. Studies have established that family support is essential for improving well-being and self-management of diabetes [2], including improving family cohesion [3]. Family environments and relations are not homogenous, and this reflected in the kind of support provided by Family Members (FMs) in the promotion of self-management practices [4, 5]. Some families view an obligation to provide support to patients is as a greatest burden [2]. However, immediate family members such as spouses and children of patients are affected by the changes in patients’ health occasioned by diagnosis and need to be empowered to offer the best support [6]. Family disruptive behaviours such as bickering about diet, exercise or medications are viewed as barriers to the patient’s effective self-management [7]. In providing support to patients, family members are expected to share certain responsibilities [8]. Families can provide support relating to driving or transporting patients to healthcare facilities for collection of medication, and consultations [8]. Family members may support patients with self-care activities such as meal preparation and consumption, exercise, intake of medication, and distribution of household chores. Additionally, families can help patients in the injection of insulin, and provision of social and emotional support [8]. It has been established that attitudes and the manner family members communicate with patients have significant effect on the psychological impact, adherence to medication, and behavioural changes relating to diet and exercise [8, 9]. It is known that social support is linked with improved self-reported health and general well-being [10]. Moreover, family support is corelated with improved coping, quality of life and glycaemic outcomes [11].

A functional family is associated with adequate support for patients [3]. Studies have reported that family members are interdependent, and care for one another in the event of health needs [12, 13]. Spouses of patients are critical in the provision of support to patients. A study involving married patients indicated that men get sufficient support from their wives, compared to what they offer [14]. However, there are spouses who do not support their partners, particularly disfunctional marriages. Persuasion among couples is regarded as efficient in promoting dietary behaviours [15]. An Indian study reported that family support was significantly related to better self-management activities [16]. Families of diabetic patients are impacted by the victim’s health and how the disease is being managed [17].

Involvement of family members in the care of patients is critical in the management and curbing of diabetes prevalence, which is skyrocketing and estimated at 537 million diagnosed persons in the world [18]. Diabetes prevalence in South Africa is highest in Africa at 11.3%, with 4.2 million diagnosed cases which is 1 in 9 adults [18]. The prevalence is projected to increase in future. Therefore, more and more patients would require family support to put the condition under control. Family support is linked with improved diabetes outcomes, whereas the lack thereof is associated with poor outcomes. Lack of family support for patients’ self-management behaviours can prevent efforts for implementation of necessary behavioural changes [19]. Conversely, absence, reduction, or loss of family support can have negative health effects on the patients [17]. Living together with people in the same household doesn’t guarantee support in diabetes management. Hence it is critical to let patient tell their lived experiences with regard to support from their family members. Therefore, this study is aimed at exploring family support in diabetes management.

## Research methodology

### Method and design

A qualitative approach and phenomenological exploratory descriptive design were applied in this study. Patients living with diabetes were individually interviewed to explore family support in diabetes management.

### Study setting

This study was conducted in eight clinics of Senwabarwana, in the Blouberg Local Municipality of the Capricorn District Municipality in the Limpopo Province, South Africa. There are several patients living with diabetes mellitus, which enabled sampling of patents meeting inclusion criteria. Diabetes prevalence in the area is on the rise, and several diagnosed patients are on treatment which they get from the primary healthcare facilities such as clinics. Senwabarwana is predominantly a rural area, and most persons residing in the area are Africans of Sepedi culture.

### Ethical considerations

Turfloop Research Ethical Committee (TREC) approved the study and allocated clearance certificate number *TREC/35/2019: PG*. Limpopo Department of Health granted permission to conduct the study (*Ref: LP 201903–007)*. All methods were performed in accordance with the Declaration of Helsinki. All respondents voluntarily gave and signed written informed consent for participation in the study. Participants were alerted about their privileges to withdraw from the study at any stage without punishment, and that their withdrawal would not affect diabetes treatment received from the facilities. None of the respondents withdrew from the study. Privacy of the respondents was maintained by interviewing patients in private consulting rooms of the clinics. Confidentiality was maintained by not capturing the real names of participants.

### Sampling and sampling size

Only outpatients diagnosed with diabetes (type 1 and type 2) and receiving treatment in clinics of Senwabarwana. Additionally, patients who had lived with diabetes for at least 6 months or more, and aged 18 years or above. A total of 8 clinics were purposively chosen because they had many patients living with diabetes within their records and receiving diabetes treatment. Therefore, it was easier to identify participants who met inclusion criteria. Subsequently, a total of 17 participants who met inclusion criteria were purposively sampled, based on data saturation.

### Data collection instrument and procedure

Data collection method adopted and applied in this study was semi-structured interviews (annexed), which involves use of open-ended questions and interview guide with sub-questions deriving from literature [20]. One-on-one interviews were conducted after obtaining informed consent from 17 participants using voice recorders and field notes for non-verbal cues observed. The interviews were conducted in Sepedi which is a dominant language in the area. The principal question for the interview was *‘Kindly describe the support from family members post-diagnosis with diabetes’.* A pilot study was conducted in non-participating clinics within Senwabarwana, before the main study, with the purpose of pre-testing the interview guide. The pilot study findings did not inform any modification on data collection instruments, and its findings not included in the main study. Data from all participants was collected by one researcher from eight clinics within Senwabarwana. Based on the responses to each question, further probing and clarity-seeking questions were asked to obtain more information. The researcher used statements such as “*may you please tell more”* or “*please let us talk more about that*” to encourage participants to give more information related to family support. Interviews with respondents took 20–40 minutes, and the data collection process was completed within 2 months. Researcher used bracketing, intuiting and reflective remarks during the interviews. For bracketing, the researcher laid aside what is known about family support in avoidance of preconceived ideas and beliefs. For intuiting, the researcher adhered to the questions in the interview guide and remained naïve for the avoidance of his own views. Lastly, for reflection, the researcher reflected on interesting remarks from respondents to encourage them to elaborate, by stating that “*so what you are actually saying is….”*

### Measures to ensure trustworthiness

Trustworthiness was ensured through credibility, transferability, confirmability and dependability as shown in Table 1.

**Table 1:** Measures of trustworthiness

Strategy	Criterion	Applicability
Credibility	Prolonged engagement	The first author worked at the only hospital in Blouberg as a dietitian, which provide service delivery through outreach to the clinics. The researcher is known in the area and also stayed in the field for an extended period to build trust and rapport with participants.
	Triangulation	Researchers used both interviews and observations in the collection of data.
	Member checks	Follow up interviews were conducted with participants after data analysis for confirmation.
Transferability	Data saturation	Data were collected until saturation was reached, which was achieved 17 participants.
	Detailed description	A detailed description of the study setting, target population and methodology for other researchers to conduct similar study in different setting.
Confirmability	Peer review	The researcher independently coded and recoded data and also developed themes. We acquired the services of an independent coder who also analysed the data. This ultimately led to a consensus meeting of the researcher and indent coder for confirmation of findings.
	Neutrality	The researcher remained neutral and was not emotional throughout the data collection and prolonged engagements.
Dependability	Full description of research methods	Full description of research methods and reporting of verbatim results makes the findings dependable.
	Reliance on data collection tools, independent coder and supervisors	The researcher relied on the voice recorder and field notes taken by an independent coder and supervisors.

### Data analysis

The recorded semi-structured interviews, conducted in Sepedi, were transcribed verbatim and translated to English, and further presented to a language interpreter. The researcher began analysing transcripts and field-notes after each data collection session. Subsequent to the researcher completion of individual data analysis, the data transcripts and field-notes were submitted to an independent coder for further analysis. The coding was done manually. Thereafter, a meeting between the researcher and independent coder was convened and agreement regarding theme and sub-themes was reached. Participants’ direct quotations are made and caught in italic format to support findings. Data was analysed using 8 Steps of Tesch’s inductive, descriptive open coding technique [21], as outlined below:

- Step 1 – Reading through the data

All verbatim transcripts were read carefully and repeatedly to get gist about data segments and its meaning. Subsequently, ideas were jotted down.

- Step 2 – Reduction of the collected data

Codes were developed based on the existence or frequency of concepts used in the verbatim transcriptions. Afterwards, topics were list and grouped together according to similarity, and those without association were clustered separately.

- Step 3 – Asking questions about the meaning of the collected data

In obtaining the meaning of data from coding, researcher asked self-questions such as “What is this about?” and “What is the underlying meaning?”

- Step 4 – Abbreviation of topics to codes

Topics from codes were abbreviated and written next to the appropriate segments of the transcription. All these codes were written in the margins of the paper.

- Step 5 – Development of themes and sub-themes

A theme and sub-themes were developed from coded data, associated texts and grouping topics.

- Step 6 – Compare the codes, topics, and themes for duplication

Re-analysis was started afresh to check for duplication, and there was no need to to refine the codes, topics, and theme.

- Step 7 – Initial grouping of all themes and sub-themes

The data belonging to each theme were assembled in one column and preliminary analysis was performed, which was followed by the meeting between the researcher, supervisors and co-coder to reach consensus on themes and sub-themes that each one had come up with independently.

- Step 8 – Recoding

There was no need for recoding.

## Results

### Demographics of study participants

Table 2 shows that 10 of the participants were males, 11 were 60 years of age and that they had more than 5 years living with diabetes.

**Table 2:** Demographic profile of participants

Participant no.	Age	Gender	Years living with diabetes	Economic status
1	60	M	Over 5 years	Pensioner
2	63	F	Over 2 years	Pensioner
3	65	M	Over 5 years	Pensioner
4	63	M	Over 10 years	Pensioner
5	64	M	5–6 years	Pensioner
6	56	F	4 years	Unemployed
7	58	M	22 years	Unemployed
8	47	M	2 years	Unemployed
9	69	M	23 years	Pensioner
10	56	F	8 months	Unemployed
11	54	F	5 years	Unemployed
12	91	F	9 months	Pensioner
13	52	F	Over 3 years	Unemployed
14	73	M	5 years	Pensioner
15	80	M	18 years	Pensioner
16	61	F	14 years	Pensioner
17	84	M	Since 90s	Pensioner

### Themes and sub-themes emerged from data analysis

Table 3 shows that 4 sub-themes emerged from the theme family support. The sub-themes are support by family members with regards to food, exercise and collection of medication, support from wives of diabetic men who lost sexual drive, Lack of support by husbands for women diagnosed with diabetes, and support from wives of diabetic men who lost income/employment.

**Table 3:** Themes and sub-themes

Theme	Sub-theme
Family support	Support by family members with regards to food, exercise and collection of medication
	Support from wives of diabetic men who lost sexual drive
	Lack of support by husbands for women diagnosed with diabetes
	Support from wives of diabetic men who lost income/employment

### Theme 1: Family support

Participants in this study indicated that their family members provided support regarding self-care activities post-diagnosis with diabetes. The following sub-themes which emerged from this theme describe the family support for patients living with diabetes.

### Sub-theme 1.1: Support by family members with regards to food, exercise and collection of medication

Diabetes treatment requires intake of medication or injection with insulin, including lifestyle modifications regarding healthy food consumption and physical activity. Participants in this study highlighted that their family members have been helpful and supportive regarding collection of medication, food preparation and physical activity. This is supported by below quotations of participants:

Participant 12: “*My family knows that I’m diabetic and are supportive. They cook for me. They let me exercise by sweeping the floor since I’m in my 90s but supervise me to ensure that I don’t overwork myself.”*

Participant 15: “*The family knows my diagnosis, and they know food which I’m supposed to eat and not to eat and are giving me accordingly.”*

### Sub-theme 1.2: Support from wives of diabetic men who lost sexual drive

Sexual dysfunction is common among patients living with diabetes. Male participants in this study indicated the presence of sexual problems and resulting in their inability to sexually satisfy their wives. However, these male participants reported that they still get support from their wives with diabetes self-care activities, as evident in the following quotation:

Participant 1: “*My wife still cooks for me and makes sure I eat food which diabetes patients are supposed to eat such as less or no fats, sugar, and salt. “.*

Participant 4: “*Although I never consulted dietitian with my wife, she has been willing to consult with me, but her work schedule prevented her; however, I relayed what I was advised to eat including the document highlighting food to eat. She always makes sure that I eat accordingly.”*

Participant 17: “*I live with my wife. She cooks for me. When she is happy, I get happy with her. When she is emotional, I just avoid her so I can avoid stress. I have erectile dysfunction and the wife understands. She is also too old; she no longer desires sex that much like before.”*

### Sub-theme 1.3: Lack of support by husbands for women diagnosed with diabetes

Female participants in this study living with their husbands indicated that they disclosed their diabetes diagnosis to their husbands, including how they should be cared for or supported. However, these female participants alluded that they receive no support from their husbands as evident in the following quotations:

Participants 10: “*My husband doesn’t fully support me, sometimes we always fight over salt-free dietary changes, and when I’m engaged and cannot manage to go to the clinic for collection of medication, he cannot collect it on my behalf. I instead request neighbors to do so, since our children are at school or work.”*

Participant 13: “*My family knows I have diabetes and gives support, however my husband was being difficult and not supportive, when I first got diagnosed with hypertension and then diabetes I was pregnant, and I was trying to prepare family meals without salt, but my husband always insulted and tortured me as if I’m the one who chose to have the disease and I got stressed and admitted..”*

### Sub-theme 1.4: Support from wives of diabetic men who lost income/employment

Male participants in this study indicated that they never consulted healthcare providers with their wives, however, they have relayed how they should be supported and cared with regards to food and other self-care practices. However, loss of income/ employment, marital conflicts started leading to unsatisfactory support from their wives. Below quotations support this sub-theme:

Participant 7: “*At my family, my wife sometimes gives me chicken feets or other fatty food while she knows very well that I’m not supposed to eat fats. When I complain, we fight, so to avoid fights, I just keep quiet and eat”.*

Participant 8 “*When I tell my wife that the food, she give me to eat will kill me, the wife will instead reply by saying if you die, you die. This treatment started when I was no longer employed, though I built her a house”.*

## Discussions

This study recognizes that family support for patients living with diabetes is crucial for better outcomes, general well-being, coping and improved health status, including prevention of complications. It was established that participants still get support from family members regarding food, exercise, and collection of medication. Diabetic men with sexual dysfunction also get similar support from wives, whilst female diabetics do not get support from their husbands. However, loss of income among diabetic men has been reported as a source of martial conflicts and unsatisfactory support from wives.

Study interviewees reported receiving support from their family members, in the form of collection of medication, and preparation and serving of food or meals. The findings of this study are consistent with an Indonesian study which reported that diabetes patients were supported at home, by family members they lived with [22]. Nutrition and exercise, including medication use or insulin injection are essential in the management of diabetes for all age groups and gender [18, 23]. Therefore, support in this regard becomes critical. It has been shown that family support to patients enhances compliance with self-care activities at home [24]. Diabetes management requires certain responsibilities from patients, which includes honouring medical appointments, adherence to medication or insulin, glucose monitoring, healthy eating and increased physical activity [25]. Inappropriate support particularly from unknowledgeable family members may be detrimental, and lead to poor diabetes outcomes. The limitation of this study is that it did not assess knowledge of the participants and their family members. Therefore, it is essential to assess knowledge of family members regarding diabetes self-care activities [26], for the purpose of determining the kind of support they are likely to give to patients. The outcomes of the assessment would inform whether to introduce community diabetes education to empower family members as essential providers of Diabetes Self-Management Support (DSMS). The DSMS is the required support for implementation and sustainability of coping skills and behaviours needed to self-manage on an ongoing basis [27], and usually provided at home by family members. Family members can help patients with meal-planning, medication reminders, glucose checking, and engaging in exercise for self-management adherence and improved well-being of both the patient and their family [28, 29]. This has been validated in this study. In African culture, supporting a family members diagnosed with diseases such diabetes can be attributed to the concept of *botho* or humanity. *Botho* is crucial philosophy of Africans, which is rooted in humanness, caring, sharing, respect, and compassion leading to happiness and peace [30, 31]. Compassion as a principle of *botho*, involves a sense and feeling of care, sympathy and concern for another person which becomes evident through showing sympathy, sharing, and helping another human being [32]. This include helping male diabetic patient experiencing sexual dysfunction.

Male study subjects’ experiences sexual problems, which is common problem among patients living with diabetes [33, 34]. Moreover, they reported receiving support from wives, despite their inability to sexually satisfy them. On the contrary, female participants in this study reported lack or unsatisfactory support from their husbands. This could be attributed to African culture wherein they used to take their wives home for care when ill. Moreover, this findings contrast a study which reported that husbands of diabetes females were prepared and willing to modify their dietary behaviours to accommodate the needs of their diabetic wives [35]. Various studies have reported that women outpace men in their responsiveness to their partner’s need for support [36, 37]. Therefore, this study also confirms that women respond positively to their husbands’ health needs compared to males. Similarly, we learned that loss of income among diabetic men has been reported as a source of martial conflicts and unsatisfactory support from wives. Therefore, this study recommends an intervention which is based or guided by *botho* philosophy for adequate diabetic management support. This could enhance the family support, which is fundamental to better outcomes.

According to this study, patients’ families assist them with meal preparation, exercise, and collection of medication. Based on the literature that is currently accessible, this study draws the conclusion that family support provided to these diabetics can result in adherence to diabetic treatment [7]. Although, the effectiveness of patient families’ support was not evaluated in this study. Therefore, it is recommended that a study be conducted to evaluate the efficacy of this family support for patients. Additionally, this study identifies knowledge of family members regarding diabetes nutrition and exercise care as critical issue, which determines the kind of support provided to patients. Therefore, it is recommended that diabetes self-care knowledge study be conducted to determine understanding of family members. Additionally, this study demonstrates that men and women react to their spouses’ medical problems in various ways. As a result, gender disparities in diabetes treatment are seen as a crucial issue that necessitates gender-biased, family-centered diabetes care.

## Conclusion

Family members of diabetic patients collect medication from healthcare facilities for patients, including cooking and serving meals to them. This could help patients adhere to medication use and dietary treatment. Wives of diabetic men outpace husbands of diabetic women in responsiveness to the health needs of their partners; diabetic men with impaired sexual function get support from their wives, whilst female patients do not get similar support from their husbands. On the other hand, male diabetics who lost income get inadequate support from spouses, which compromises diabetes management and adherence to treatment. This study identifies gender disparities in provision of spousal support as crucial issues requiring an urgent attention, hence gender-biased family-centred diabetes care is recommended.

## Data Availability

This article is based on the data collected from patients living with diabetes in Senwabarwana, Blouberg Municipality in Limpopo Province, South Africa. The data generated or analysed during the current study is not publicly available, but requested from Mphasha.

## Abbreviations

DSMS: Diabetes Self-Management Support
TREC: Turfloop Research Ethical Committee
